# Age-Related Eye Disease and Participation in Cognitive Activities

**DOI:** 10.1038/s41598-017-18419-2

**Published:** 2017-12-21

**Authors:** Melanie Varin, Marie-Jeanne Kergoat, Sylvie Belleville, Gisele Li, Jacqueline Rousseau, Marie-Hélène Roy-Gagnon, Solmaz Moghadaszadeh, Ellen E. Freeman

**Affiliations:** 1 School of Epidemiology and Public Health, University of Ottawa, Ottawa, Canada; 2 Centre de Recherche, Institut universitaire de gériatrie de Montréal, Montréal, Canada; 3 Centre de Recherche, Hôpital Maisonneuve-Rosemont, Montréal, Canada; 4 Department of Ophthalmology, Université de Montréal, Montréal, Canada; 5 Ottawa Hospital Research Institute, Ottawa, Canada

## Abstract

Studies have found a benefit to living a cognitively active life in older age. Our goal was to quantify participation in cognitively stimulating activities in adults with and without age-related eye disease. We conducted a cross-sectional hospital-based study in Montreal, Canada of older adults (n = 303) having either age-related macular degeneration (AMD) (n = 96), glaucoma (n = 93), or normal vision (n = 114). To be eligible, the AMD group had to have bilateral late stage AMD with a better eye visual acuity of 20/30 or worse. The glaucoma group had to have a diagnosis of bilateral primary open-angle glaucoma with visual field mean deviation <  = −4 dB in their better eye. Further inclusion criteria included age ≥ 65 and a Mini-Mental State Exam Blind score ≥ 10. Cognitive activities were measured using the Victoria Longitudinal Study Activity Questionnaire. Linear regression was used. Patients with AMD (β = −4.2, 95% confidence interval (CI) −6.0, −2.4) and glaucoma (β = −1.8, 95% CI −3.3, −0.3) participated in fewer cognitive activities per month compared to those with normal vision after adjusting for age, sex, education, diabetes, number of comorbidities, cognition, and cataract. People with AMD and glaucoma participated in fewer cognitive activities, which could put them at risk for future cognitive impairment.

## Introduction

Many observational studies have suggested the cognitive benefits of living an active, cognitively stimulating life^1–3^. For example, older adults who were more likely to engage in activities like reading, playing games, and going to museums were 33% less likely to develop Alzheimer’s disease^1^. Researchers have found that the variety of activities that people participate in has been shown to be predictive of subsequent cognitive decline^3^. Research by Carlson *et al*. found that each additional activity per month was associated with an 11% lower risk of incident cognitive impairment as measured using the Mini-Mental State Exam (MMSE)^3^. However, it may be difficult for people with age-related eye disease to participate in a wide variety of cognitive activities. Reduced cognitive activity participation may put older adults with eye disease at risk for cognitive decline and may explain why they seem to have lower cognitive scores^4–7^. To our knowledge, no prior studies have quantified the frequency of cognitive activities in people with and without eye disease.

Our primary objective was to determine whether people with age-related macular degeneration (AMD) or glaucoma, two of the most common causes of visual impairment in older adults, participate in fewer cognitive activities compared to older adults with normal vision. We chose an activity questionnaire developed by cognitive aging researchers that focuses on activities that are cognitively stimulating and that quantifies the time people spend doing various activities^8^. We hypothesized that patients with AMD and glaucoma would have reductions in total activities compared to people with normal vision. Our secondary objective was to determine whether any reductions in activities in those with AMD or glaucoma were due solely to specific subtypes of activities (e.g. physical, social, hobbies, novel information processing, etc). We hypothesized that patients with AMD would be affected across all activities but would have the lowest scores on activities using novel information processing due to their difficulties with reading^9^. We hypothesized that glaucoma patients would generally be less affected than AMD patients except for physical activities due to difficulty with balance^10,11^.

## Methods

### Study Design and Population

Participants in this hospital-based cross-sectional study were recruited from the Hôpital Maisonneuve-Rosemont (HMR) (Montreal, Quebec) ophthalmology clinics from 2016–2017. In total, we recruited 303 people in one of 3 groups that met the following inclusion criteria: 1) those with late stage AMD (geographic atrophy or neovascular disease) in both eyes with a better eye visual acuity of 20/30 or worse, 2) those with a diagnosis of primary open-angle glaucoma in both eyes with visual field mean deviation worse than or equal to −4 dB in their better eye, 3) and those who had normal visual acuity and visual field (better than 20/30 in both eyes and visual field mean deviation better than −3 dB in both eyes). The normal vision group did not have early or late AMD, glaucoma, or suspected glaucoma. The leading conditions for which the normal vision group were being seen included early stages of cataract, early stages of diabetic retinopathy, and posterior vitreous detachment. Exclusion criteria included if patients were under 65, if they had a score less than 10 on the Mini-Mental State Examination (MMSE) Blind Version, which is an indicator of cognitive impairment^12^, or if they had eye surgery or other surgery in the last 2 months. Participants who had had surgery could participate after the 2 months had passed under the assumption that their activity levels and mood would be similar to pre-surgery levels^13^. Approval from the Ethics Committee at Maisonneuve-Rosemont Hospital was obtained. Written informed consent was acquired from each person. This research followed the tenets of the Declaration of Helsinki. The datasets generated during and/or analysed during the current study are available from the corresponding author on reasonable request.

### Data Collection

#### Vision and Eye Disease

The Early Treatment of Diabetic Retinopathy Study (ETDRS) visual acuity chart was used to measure binocular visual acuity at 2 meters wearing the normal prescription for distance correction^14,15^. Scores were converted to log of the minimum angle of resolution (logMAR). Visual field was measured using the Humphrey frequency doubling technology (FDT) perimeter full-threshold N-30 testing in each eye^16^. The medical chart was reviewed for details on the patient’s eye disease (e.g. date of diagnosis, type of AMD) and any co-existing eye disease (lens opacity).

#### Cognitive Activities

All questionnaires were interviewer-administered in a face-to-face manner. Cognitive activities were measured with the Victoria Activity Questionnaire, which is a 70-item survey assessing older adults’ participation in various cognitive activities over the past two years^8^. This test was found to have very good test-retest reliability^17^. Examples of activities included in the questionnaire are: gardening, exercise, preparing meals, doing housework, attending church, going shopping, giving a dinner party, engaging in sculpting/painting, working on crossword puzzles, writing letters or email, watching documentaries on television, etc. Each activity was assigned to one of 6 subtypes as determined by prior research (e.g. physical activities, self-maintenance activities, social activities, hobbies and home maintenance, novel information processing, and passive information processing)^8^. Respondents were asked to rate the frequency of their participation for each item based on a 9-point scale (never, less than once a year, about once a year, 2 or 3 times a year, about once a month, 2 or 3 times a month, about once a week, 2 or 3 times a week, and daily). The total number of activities completed at least monthly was then used as the primary outcome^3^, while the subtypes of activities done at least once per month were used as secondary outcomes.

#### Demographics and Health

Data were collected on each participant’s age, sex, and number of years of formal education. The presence of a current lens opacity was taken from each participant’s medical chart. Participants were asked about a physician diagnosis of 13 chronic conditions (e.g. chronic obstructive pulmonary disorder, diabetes, arthritis). The MMSE Blind Version, which is an adaptation of the Mini Mental State Examination for adults with impaired vision, was administered^12^. Depressive symptoms were evaluated by the Geriatric Depression Scale Short Form^18^.

### Statistical analysis

To compare our three groups with and without eye disease, ANOVA and chi-square tests were used to assess whether there were any global differences in the number of activities done at least once per month. The normality of the total cognitive activity levels and each sub-group of activity levels was examined using standardized normal probability plots. To test whether total cognitive activity levels were different between the three groups, ANOVA was used. To test whether sub-groups of activity levels were different between the three groups, the Kruskal-Wallis test was used due to a lack of normality. For total activities, our primary outcome, linear regression was used to adjust for variables that were considered as potential confounders of the relationship between eye disease and activity levels like age, sex, education, diabetes, comorbidity level, cognitive status, and lens opacity. For the subtypes of activities, linear regression with robust variance estimation (Huber/White/sandwich estimator) was used due to the non-normality of the distributions.

## Results

Of those patients that were thought to be eligible for age and diagnosis based on a review of the medical chart (n = 751), 401 (53%) agreed to participate, 294 (39%) refused, and 56 (7%) were not capable of answering by themselves. There were no statistically significant differences in age or sex between those who participated and those who did not (data not shown). Of the 401 who agreed to participate, 315 completed data collection, 48 did not meet the remaining eligibility criteria, and 38 did not complete data collection.

Demographic, vision, and health characteristics of the cohort can be found in Table 1. There were 303 Caucasian participants, of whom 96 had AMD, 93 had glaucoma, and 114 had normal vision. The groups with AMD and glaucoma were older, had less education, had worse visual acuity, worse visual field, worse cognition, and had higher depression scores. Furthermore, the AMD group contained more women and had more comorbidities.

**Table 1:** Description of the study population.

	AMD Mean (SD) or % n = 96	Glaucoma Mean (SD) or % n = 93	Normal Vision Mean (SD) or % n = 114	P-value*
Age, Years	83.6 (7.0)	77.5 (8.0)	72.8 (6.0)	<0.001
Sex				
Men	30.2%	40.9%	48.3%	0.003
Women	69.8%	59.1%	51.8%	
Education, Years	10.0 (4.4)	11.1 (4.2)	12.5 (4.0)	<0.001
Binocular visual acuity, logMAR	0.57 (0.40)	0.21 (0.25)	0.05 (0.06)	<0.001
Visual field in better eye, MD in dB	−4.2 (4.0)^†^	−9.0 (7.1)	0.8 (2.4)	<0.001
Geriatric Depression Scale Score (max: 15)	3.5 (2.7)	2.8 (2.9)	2.1 (2.1)	<0.001
Mini-Mental State Exam Blind Score (max: 22)	19.7 (2.6)	20.0 (2.4)	20.9 (1.4)	<0.001
No. of comorbidities	3.2 (1.7)	2.7 (1.7)	2.7 (1.5)	0.029
Lens opacity in eye	22.0%	18.9%	29.2%	0.336

SD = standard deviation, MD = mean deviation, dB = decibels, logMAR = log minimum angle of resolution.

*P-value derived from ANOVA or chi-square test.

### Unadjusted results

As shown in Table 2, the AMD and glaucoma groups performed fewer physical activities (P < 0.001), social activities (P = 0.020), hobbies/home maintenance activities (P < 0.001), passive information processing activities (P = 0.006), and novel information processing activities (P < 0.001) compared to the normal vision group. The glaucoma group performed fewer self-maintenance activities than the normal vision group (P = 0.017). The largest difference was seen in the novel information processing group where the AMD group (median = 5.0, interquartile range (IQR) = 4.0) performed a median of 4 fewer activities per month than the normal vision group (median = 9.0, IQR = 4.0). The glaucoma group (median = 7.0, IQR = 4.0) participated in a median of 2 fewer novel information processing activities than the normal vision group (median = 9.0, IQR = 4.0). In terms of the total number of activities performed at least once per month, both the AMD (mean = 17.6, SD = 5.9) and glaucoma (mean = 22.5, SD = 6.6) groups did fewer total activities per month compared to older adults with normal vision (mean = 25.5, SD = 5.3) (P < 0.001).

**Table 2:** Description of the sum of activities done at least once per month overall and by subtype of activity.

	Physical Activities Median (IQR)	Self-Maintenance Activities Median (IQR)	Social Activities Median (IQR)	Hobbies and Home Maintenance Median (IQR)	Passive Information Processing Median (IQR)	**Novel Information Processing Median (IQR)**	**Total Activities Mean (SD)**
Eye Disease Group							
Normal Vision	2.0 (1.0)	5.0 (1.0)	2.0 (2.0)	2.0 (1.0)	4.0 (1.0)	9.0 (4.0)	25.5 (5.3)
AMD	1.0 (2.0)	5.0 (1.0)	1.0 (1.0)	0.0 (1.0)	4.0 (2.0)	5.0 (4.0)	17.6 (5.9)
Glaucoma	1.0 (1.0)	4.0 (1.0)	1.0 (1.0)	1.0 (2.0)	4.0 (2.0)	7.0 (4.0)	22.5 (6.6)
Range	0–6	0–6	0–5	0–7	0–6	0–16	3–40
P-value*	<0.001	0.017	0.020	<0.001	0.006	<0.001	<0.001

IQR = inter-quartile range, SD = standard deviation.

*P-value derived from Kruskal-Wallis test (activity subgroups) or from ANOVA (total activities).

### Adjusted results

In Table 3, linear regression was used to adjust for demographic and health variables. Older adults in the AMD group performed 4.19 fewer total cognitive activities per month compared to older adults in the normal vision group (95% CI −5.96, −2.43). The glaucoma group performed 1.8 fewer activities per month compared to the normal vision group after adjustment (95% CI −3.34, −0.26). Other variables that were associated with total cognitive activities included age (β = −0.23, 95% CI −0.32, −0.13), education (β = 0.33, 95% CI 0.16, 0.49), and score on the MMSE-Blind (β = 0.38, 95% CI 0.06, 0.70). As for the subtypes of activities, after adjustment, only AMD was associated with reduced physical activities (β = −0.49, 95% CI −0.78, −0.20), hobbies and home maintenance activities (β = −0.59, 95% CI −1.02, −0.16), and novel information processing activities (β = −2.00, 95% CI −2.90, −1.11). Neither AMD nor glaucoma were associated with social, self-maintenance, or passive information processing activities after adjustment (Appendix).

**Table 3:** Linear regression models of relationships between age-related eye disease and total cognitive activities and subtypes of activities.

Co-variates	Model 1 Total Cognitive Activities β 95% CI	Model 2 Physical Activities β 95% CI	Model 3 Hobbies and Home Maintenance β 95% CI	**Model 4 Novel Information Processing β 95% CI**
Eye disease group				
Normal vision	0.00	0.00	0.00	0.00
AMD	−4.19 −5.96, −2.43*	−0.49 −0.78, −0.20*	−0.59 −1.02, −0.16*	−2.00 −2.90, −1.11*
Glaucoma	−1.80 −3.34, −0.26*	−0.26 −0.53, 0.02	−0.05 −0.49, 0.40	−0.67 −1.45, 0.12
Age	−0.23 −0.32, −0.13*	−0.01 −0.03, 0.00	−0.01 −0.04, 0.01	−0.09 −0.14, −0.04*
Female sex	0.13 −1.17, 1.44	−0.13 −0.36, 0.10	−0.12 −0.46, 0.23	0.31 −0.37, 0.99
Education	0.33 0.16, 0.49*	−0.00 −0.03, 0.03	0.07 0.03, 0.12*	0.29 0.21, 0.37*
Number of Comorobidities	−0.02 −0.42, 0.38	−0.00 −0.07, 0.07	−0.04 −0.15, 0.07	−0.06 −0.26, 0.14
Diabetes	−1.42 −2.94, 0.10	−0.20 −0.48, 0.07	0.05 −0.33, 0.42	−0.50 −1.26, 0.26
Cataract	0.57 −0.91, 2.05	0.07 −0.18, 0.31	0.22 −0.14, 0.58	−0.05 −0.73, 0.63
MMSE-Blind Score	0.38 0.06, 0.70*	0.06 0.02, 0.11*	−0.01 −0.10, 0.08	0.12 −0.07, 0.30

*P < 0.05.

## Discussion

Our study is the first to quantify the number of activities done by people with eye disease compared to people without eye disease. Patients with AMD did 4.19 fewer cognitively stimulating activities per month than older adults with normal vision. Most affected for AMD patients were physical activities, hobbies and home maintenance activities, and novel information processing activities, which includes activities like reading books, doing crossword puzzles, attending films or concerts, and doing volunteer work. This fit with our hypothesis that AMD patients would be affected across a variety of activities but that activities requiring novel information processing would be most affected. Patients with glaucoma were less affected although they still did 1.8 fewer activities per month than those with normal vision. Their scores were lowest for the subtypes that included physical activities and novel information processing activities although neither subtype reached statistical significance.

The loss of visual acuity in AMD patients can lead to great difficulty with reading, driving, and exercising, which are abilities that are required for many of the activities that we examined^9,19,20^. Visual field loss due to glaucoma can also make it difficult to engage in activities as it has been associated with limited mobility, reading difficulties, driving modification and cessation, and crash^21–25^. Low vision rehabilitation can make these activities more feasible but many older adults do not participate^9,26,27^.

Performing fewer cognitive activities could put patients with age-related eye diseases like AMD and glaucoma more at risk for cognitive impairment^1–3^. Indeed, research by Carlson *et al*. found that each additional activity per month performed was associated with an 11% lower risk of incident MMSE impairment^3^. Many studies have documented reduced cognitive function in those with age-related eye disease but other studies have not^4–6,28–30^. Longitudinal studies are needed to determine whether adults with age-related eye disease have worse cognition because of performing fewer cognitive activities.

Our study is the first to quantify the frequency of activity levels as measured using a cognitive aging questionnaire in people with and without eye disease, and in particular, to look at variety of activities. However, other studies have examined difficulty with performing activities using questionnaires developed to assess visual disability. For example, Desrosiers *et al*. measured visual disability using the Assessment of Life Habits questionnaire which measures 77 life habits, their degree of difficulty, and assistance used^31^. A 10-level score combined these two ratings. They found that 64 older adults with visual impairment recruited from a low vision rehabilitation clinic had lower scores than 64 people who had participated in an unrelated population-based study (P < 0.001). Lamoureux *et al*. measured a person’s difficulty with doing activity levels using the Impact of Vision Impairment questionnaire, which comprises 32 items, in 319 people attending a low vision rehabilitation clinic and found that worse distance visual acuity was related to worse scores on several activity domains (P < 0.01)^32^. These previous studies have concluded that people with eye disease have *more difficulty* performing various activities whereas our study conclusion is that people with eye disease are *less cognitively active*. Less cognitive activity may put people with eye disease at greater risk of subsequent cognitive decline^1,2^.

The strengths of this study included the large numbers of people with AMD or glaucoma and having a comparison group with normal vision that was recruited from the same clinic. Our study was novel in using a questionnaire designed by cognitive aging researchers that focused on activities that are cognitively stimulating. A limitation is that our study is cross-sectional so we cannot establish the temporality of the onset of vision loss and any reduction in the level of activities. Also, our refusal rate was 39% which is typical for a hospital-based study. We did not detect any statistically significant differences in age or sex between those who agreed to be in the study and those who refused so we think that the threat of selection bias is very low. Finally, these results may only be generalizable to AMD and glaucoma patients with bilateral disease who meet our acuity and visual field inclusion criteria.

Patients with AMD and glaucoma participated in fewer cognitive activities compared to older adults with normal vision, potentially putting them at risk for cognitive impairment. Efforts to increase their activity participation rate via low vision rehabilitation, occupational therapy, assistance from family and friends, and community programs should be explored^33^.

## Electronic supplementary material

Appendix
