# Supplementary material for: Age-Related Eye Disease and Participation in Cognitive Activities
Source: Sci Rep. 2017 Dec 21;7:17980. doi: 10.1038/s41598-017-18419-2 (PMC5740122; doi:10.1038/s41598-017-18419-2)
Supplement: Supplementary file 1 — Appendix [file 41598_2017_18419_MOESM1_ESM.pdf]

Age-Related Eye Disease and Participation in Cognitive Activities

Melanie Varin[1], Marie-Jeanne Kergoat[2], Sylvie Belleville[2], Gisele Li[3,4], Jacqueline Rousseau[2], Marie-Hélène Roy-Gagnon[1], Solmaz Moghadaszadeh[3], Ellen E. Freeman[1,3,4,5]

[1] School of Epidemiology and Public Health, University of Ottawa; [2] Centre de Recherche, Institut universitaire de gériatrie de Montréal; [3] Centre de Recherche, Hôpital Maisonneuve-Rosemont, Montréal; [4] Department of Ophthalmology, Université de Montréal; [5] Ottawa Hospital Research Institute, Ottawa; Canada

MV contributed to the analysis, the interpretation of the data, and the drafting the manuscript. MJK, SB, GL, JR, and MHRG contributed to the interpretation of the results and the revision of the manuscript. SM collected the data, interpreted the data, and revised the manuscript. EEF contributed to the acquisition, analysis, and interpretation of the data and the revision of the manuscript. All authors gave final approval for the version to be published and agree to be accountable for all aspects of the work.

Funded by a grant from the Canadian Institutes of Health Research (MOP 133560)

Conflict of Interest: No competing financial relationship exists for any author

Word Count: 2,269

Corresponding Author:
Ellen Freeman, School of Epidemiology and Public Health, University of Ottawa, Ottawa, Canada, eefreeman@gmail.com, 613-562-5800 x8439

Appendix: Additional linear regression models of relationships between age-related eye disease and cognitive activities

	**Self-Maintenance Activities**		**Social Activities**		**Passive Information Processing Activities**	
	**β**	**95% CI**	**β**	**95% CI**	**β**	**95% CI**
Eye disease group						
Normal vision	0.00		0.00		0.00	
AMD	-0.24	-0.62, 0.13	-0.22	-0.57, 0.12	-0.13	-0.51, 0.25
Glaucoma	-0.27	-0.59, 0.05	-0.24	-0.56, 0.08	-0.10	-0.47, 0.26

*Adjusted for age, sex, education, number of comorbidities, diabetes, cataract, MMSE-Blind score
